# Sanitary Convention

**Published:** 1882-10-20

**Authors:** 


### *SANITARY CONVENTION.*

We are in receipt of a circular sent by Wm. H. Newell, M.D., Corresponding Secretary, and signed by a number of distinguished gentlemen and prominent members of State Boards, addressed to the Secretaries of the several State Boards in the United States, in which they say:

"The undersigned would earnestly request your Board to appoint one Commissioner and an Alternate Commissioner to meet Commissioners appointed by the National and various State Boards of Health of the United States, together with Commissioners appointed by the American Public Health Association, said Comissioners to assemble at Indianapolis, Indiana, on Wednesday, October 18th, 1882, at 9 o'clock, A. M., to take into consideration the question as to the best course to be pursued, which may result in holding a National Medical and Sanitary Exhibition in the year 1883.

"You will oblige by informing us at your earliest convenience of the action taken by your Board of Health in the matter."

